# A simple method using *ex vivo* culture of hair follicle tissue to investigate intrinsic circadian characteristics in humans

**DOI:** 10.1038/s41598-017-07268-8

**Published:** 2017-07-28

**Authors:** Ai Yamaguchi, Ritsuko Matsumura, Takashi Matsuzaki, Wataru Nakamura, Koichi Node, Makoto Akashi

**Affiliations:** 1 The Research Institute for Time Studies, Yamaguchi University, 1677-1 Yoshida, Yamaguchi, 753-8511 Japan; 2 Department of Biological Science, Shimane University, 1060 Nishikawatsu-cho, Matsue, 690-8504 Japan; 3 Department of Oral-Chrono Physiology, Nagasaki University, 1-7-1 Sakamoto, Nagasaki, 852-8588 Japan; 4 Department of Cardiovascular Medicine, Saga University, 5-1-1 Nabeshima, Saga, 849-8501 Japan

## Abstract

Almost all organisms maintain a circadian clock from birth to death to synchronize their own physiology and behavior with the earth’s rotation. Because the *in vivo* evaluation of human circadian characteristics is labor-intensive, *in vitro* or *ex vivo* approaches could provide advantages. In this study, to enable the simple and non-invasive evaluation of autonomous circadian oscillation, we established a method for monitoring clock gene expression by performing *ex vivo* culture of whole hair root tissue. This method is extremely simple and imposes little burden on subjects. Results obtained using *Cryptochrome*-deficient mice support that circadian period length in hair tissue correlates with intrinsic period length observed in physiology and behavior. We then applied this method to old-old subjects with severe dementia, who showed abnormal circadian behavior, and found that their peripheral clocks autonomously oscillated in a manner similar to those of healthy or younger subjects, indicating that the effect of cellular senescence on the autonomous clock oscillator is limited at least in some cell types. Although further validation may be required, the hair tissue-based culture assay would be a tool to investigate intrinsic circadian characteristics in humans.

## Introduction

Almost all living organisms exhibit circadian rhythms in physiology and behavior, which are driven by the internal circadian clock^1, 2^. The circadian clockwork consists of cell-autonomous and clock gene-driven negative feedback loops of transcription^3, 4^. The transcriptional feedback loops generate circadian expression of a wide range of numerous genes^5, 6^, which in turn leads to circadian oscillation in diverse physiological processes^7, 8^.

Because the *in vivo* evaluation of individual intrinsic circadian characteristics in humans, either with constant routine or forced desynchrony protocols, is expensive and labor-intensive, evaluation of circadian characteristics by *in vitro* or *ex vivo* assays could present important advantages. Among efforts to realize this, Brown and colleagues reported an interesting method based on human epidermal skin biopsy^9^ which is useful for evaluating autonomous oscillation performance of clock gene expression *in vitro*. Importantly, although fibroblast circadian characteristics are not direct markers of physiological circadian characteristics, a few research groups have concluded that circadian characteristics in skin-derived fibroblasts reflects individual circadian preference, including chronotype^9, 10^. However, epidermal skin biopsy is too invasive for elderly or non-healthy subjects, and cell dispersion or cell growth steps might potentially result in changing the original circadian characteristics.

In this study, to enable the simple and non-invasive evaluation of autonomous circadian oscillation performance, including intrinsic period length, we established a method to monitor clock gene expression in real time by performing *ex vivo* culture of whole hair root tissue. An age-related shortening of circadian period has been hypothesized to account for the circadian phase advance and early-morning awakening observed frequently in the elderly. We then applied this method to investigate the effect of cellular senescence on autonomous circadian gene expression in humans, who have a much longer lifespan than model organisms such as mice. To do this, we focused on old-old subjects with severe dementia who showed abnormal circadian behavior, and then used this method to investigate the correlation between disrupted circadian behavior and peripheral circadian characteristics.

## Materials and Methods

### Subjects

Healthy subjects and old-old subjects with severe dementia who showed abnormal circadian behavior were recruited. This study was conducted in accordance with the Declaration of Helsinki and was approved by the institutional review boards of Yamaguchi University and Dokkyo Medical University. Informed consent was obtained from each subject or subject’s family.

### Adenovirus vector construction

Adenoviruses carrying the *luciferase* gene driven by circadian promoter/enhancer elements controlling the *hBmal1*, *mPer2*, and *hPer3* clock genes were constructed as follows. The transcription-regulatory region (−4799 - +171) of the human *Period3* gene was excised from a BAC clone (RP3-467L1) using Xba I and Xho I, and subcloned into the pGL3 basic vector (Promega). The resulting vector was digested using Kpn I and self-ligated to produce *hPer3* (−3124 - +171) -pGL3. In addition to *hPer3*, the previously constructed vectors, *mPer2* (−2811 - +110) -pGL3 and *hBmal1* (−3465 - +57) -pGL3, were used for adenovirus vector construction^11, 12^. Together with the luciferase gene, the transcription-regulatory region of these three genes was excised from each pGL3 vector, and the inserts were subcloned into the pENTR-1A vector (Thermo Fisher Scientific). The restriction enzymes used for digestion of the pGL3 vectors were as follows: *hPer3–luc*, Asp718 (Kpn I) and Sal I; *mPer2–luc*, Asp718 (Kpn I) and Sal I; *hBmal1–luc*, Sal I (two restriction sites, one at the 3′ end of the SV40 late poly(A) signal sequence and one inside *hBmal1*). The resulting vectors were *hPer3* (−3124 - +171) -*luc*-pENTR-1A, *mPer2* (−2811 - +110) -*luc*-pENTR-1A and *hBmal1* (−1683 - +57) -*luc*-pENTR-1A. These entry vectors were deposited into the RIKEN BioResource Center (RDB15084, RDB15083 and RDB15085, respectively). Before introduction into a destination vector, each pENTR-1A vector was transfected into NIH3T3 fibroblasts, synchronized by a dexamethasone shock (50 nM), and circadian bioluminescence rhythms were confirmed by real-time monitoring with a photomultiplier tube in the presence of luciferin. pENTR-1A inserts were introduced into the pAd/PL-DEST vector using LR recombination. After Pac I digestion, the resulting pAd/PL-DEST vectors were transfected into 293A cells to produce an adenoviral stock. After amplification, this adenoviral stock was used to express circadian-driven luciferase in hair follicle tissue. The recombinant adenoviruses were generated using the ViraPower Adenoviral Gateway Expression Kit (K4940-00, Thermo Fisher Scientific), according to the manufacturer’s instructions. An adenovirus carrying the *luciferase* gene driven by the CAG promotor was purchased from the RIKEN bioresource center^13^. Adenovirus stocks (1.24–1.47 × 10^9^ infectious units/mL, HEK293A) were added to culture medium at a 1:20 dilution.

### *Ex vivo* culture

Plucked facial or scalp hairs whose root surface was almost or fully covered with hair follicle cells were used for culture experiments. The experimental procedure for monitoring autonomous oscillation performance in clock gene expression is as follows: pluck a few strands of facial or scalp hair whose root surface is enriched with cells; immerse the whole tissue into DMEM (Sigma Aldrich, U.S.A) supplemented with 0.035% sodium bicarbonate, 10 mM HEPES, 4.5 g/l D-Glucose, 1% penicillin/streptomycin, 1 mM L-Glutamin, and 1 mM sodium pyruvate; maintain the sample in a keep-warm bag without CO2 during the transport from nursing homes to laboratories for approximately 10 h; transfer the tissue to the medium containing adenovirus and incubate for 22–24 h at 36.5 °C without dispersing cells; to avoid floating fix the hair shaft on the bottom of a 35 mm culture dish with silicone (KS-64, Shin-Etsu, Japan); and cover the immobilized tissue with luciferin (0.1 mM)-containing DMEM (Nacalai, Japan) supplemented with antibiotics and incubate it at 36.5 °C with 5% CO2. Culture medium in all experiments contained phenol red except those for Fig. 3E, which examined the effect of the presence or absence of phenol red on circadian period length. Adenoviral infection can elicit circadian oscillations in bioluminescence independently of DEX treatment; therefore, DEX treatment was excluded to simplify experimental procedures and to reduce cellular stress in experiments using adenovirus-infected human hair tissue, except for those shown in Fig. 3C and D. In contrast, all experiments using mouse hair tissue (Figs 4 and 5), which were not infected with adenovirus, were treated with DEX. Bioluminescence was measured in real time with a photomultiplier tube (LM2400; Hamamatsu, Japan) or a luminescence microscope optimized for single-cell imaging (LV200; Olympus, Japan).

### Data analysis

The data sets were detrended by subtracting the 24-h running average from the raw data. We calculated circadian robustness, circadian phase (angle), and circadian period length using the software Cosinor provided by Dr. Refinetti. Except for Fig. 2C and D, oscillation data were considered reliable when both clear circadian oscillation persisted for ≥3 days and circadian robustness was 70% or more.

### Animals

*Per2::luc* knock-in mice, *Bmal1-Eluc* transgenic mice and *Cryptochrome* knockout mice were a kind gift from Dr. Joseph Takahashi, Dr. Yoshihiro Nakajima and Dr. Takeshi Todo, respectively^14–16^. Mice were bred and maintained on a 12-hour light-dark (LD) cycle (lights on at 9:00 A.M.) and allowed *ad libitum* access to food and water. All protocols for animal experiments were approved by the Animal Research Committee of Yamaguchi University. Animal studies were performed in compliance with the Yamaguchi University Animal Care and Use guidelines.

### Isolation and culture of mouse whisker follicles

Mouse whisker follicles were isolated by microdissection using methods previously described^17^. Briefly, after euthanasia, the both left and right mystacial pads were removed from mice, washed vigorously two times with 70% ethanol and three times with phosphate-buffered saline (PBS) and then placed in Dulbecco’s modified Eagle’s medium (DMEM, Nacalai, Japan) supplemented with penicillin and streptomycin. Individual whisker follicles were carefully dissected under a dissecting microscope. Considerable care was taken to remove surrounding connective tissue but not to damage hair follicles. Whisker follicles were isolated and transferred on to a plastic Petri dish containing fresh DMEM. Hair cycle stages of whisker follicles were classified as anagen or catagen by morphology of the hair bulb and relative length of the hair shaft according to previous reports^18^. In mouse whisker follicles, termination of hair growth in the previous hair cycle and initiation of hair regeneration in the subsequent cycle partially overlap, therefore telogen is not obvious.

To avoid floating, the hair shaft was fixed on the bottom of a 35 mm culture dish with silicone (KS-64, Shin-Etsu, Japan), and the immobilized hair tissue was then covered with 0.1 mM luciferin-containing DMEM supplemented with antibiotics and incubated at 35 °C with 5% CO2. After 2-day pre-culture, circadian synchronization was performed with 100 nM dexamethasone (DEX) for a period of 2 h, and bioluminescence was measured in real time with a photomultiplier tube (LM2400; Hamamatsu, Japan). Data sets were detrended by subtracting the 24-h running average from raw data.

### Information on dementia subjects

Subject A was an 87-year-old female diagnosed with Alzheimer’s disease, and had a Mini-Mental State Examination (MMSE) score of 0 points and a Functional Assessment Staging (FAST) of 7(e). Subject C was an 86-year-old male diagnosed with frontotemporal dementia, and had an MMSE score of 12 points and a FAST of 7(c). Subject D was a 94-year-old female diagnosed with frontotemporal dementia, and had an MMSE score of 0 points and a FAST of 7(c). Subject E was an 88-year-old female diagnosed with vascular dementia, and had an MMSE score of 2 points and a FAST of 7(c).

## Results

### Development of a non-invasive and simple method for examining autonomous oscillation performance in peripheral clock gene expression

**Figure 1:**
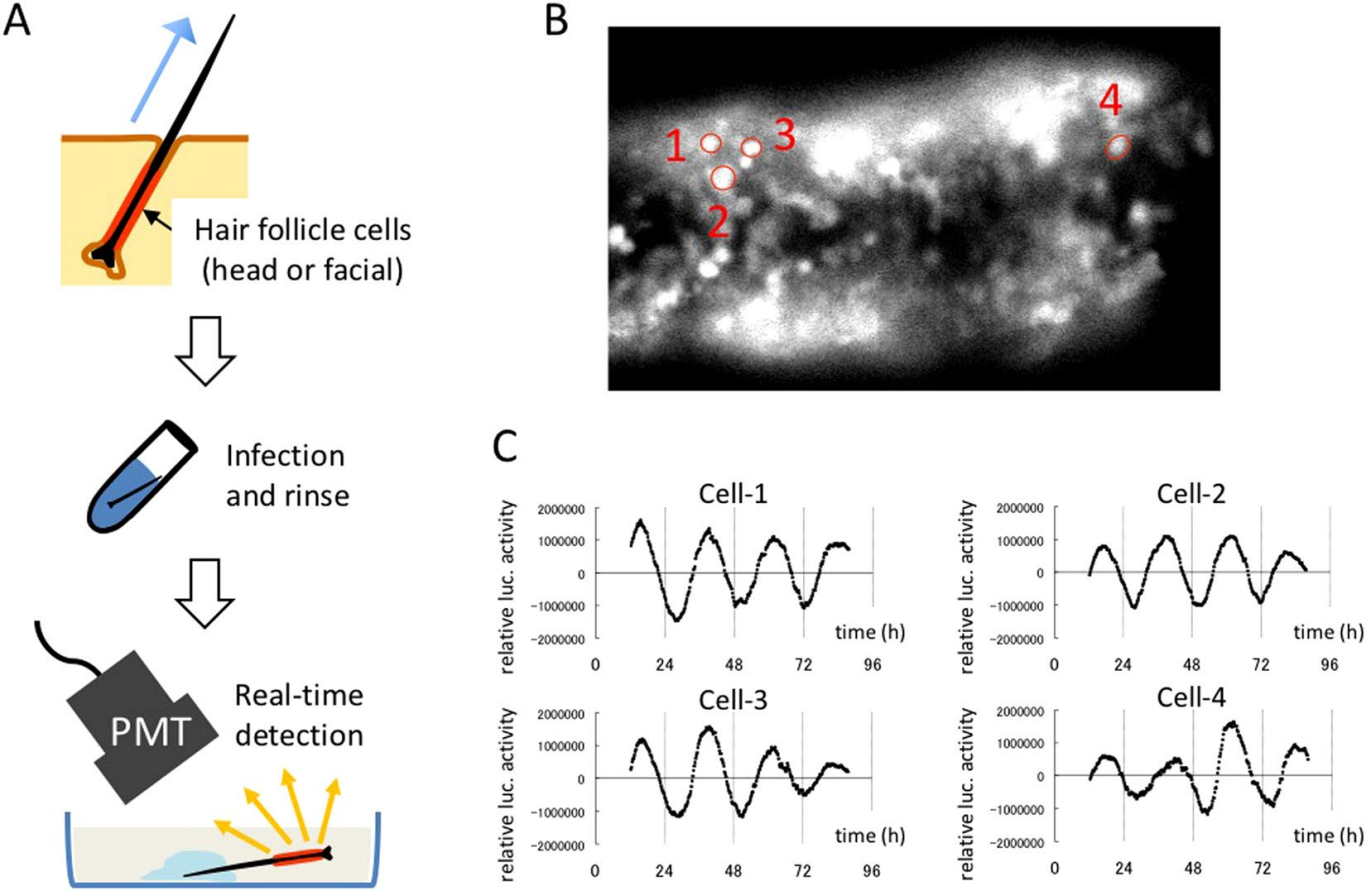
Development of a non-invasive and simple method for examining autonomous oscillation performance in peripheral clock gene expression. (**A**) A schematic representation of experimental procedures for examining autonomous oscillation performance in peripheral clock gene expression using hair follicle cells. (**B**) A representative image showing successful infection of an adenovirus carrying *Per2-luc* to a hair root. The image was obtained with a luminescence microscope optimized for single-cell imaging. Red circles surrounding individual cells indicate ROI (region of interest), which was used for quantification of signal intensity in C. (**C**) Bioluminescence was continuously measured in real time, and the intensity was integrated for 15 min at intervals of 15 min. Quantification of the intensity from each cell was performed on successive images. Data sets were detrended by subtracting the 24-h running average from the raw data.

Given that the previously reported skin biopsy method was deemed invasive for old or unhealthy subjects, we established a new method based on *ex vivo* culture of whole hair root tissue. Adenoviruses carrying the *luciferase* gene driven by circadian promoter/enhancer elements controlling the *Bmal1*, *Per2*, and *Per3* clock genes were constructed and used for gene transfer. The experimental procedure is extremely simple (Fig. 1A): pluck a few strands of facial or scalp hair whose root surface is enriched with cells, immerse the whole tissue into culture medium containing adenovirus overnight without dispersing cells, replace this medium with luciferin-containing medium, and then place the sample into a device equipped with photomultiplier tubes (PMT) to monitor clock gene expression as bioluminescence in real time. We were able to confirm successful infection at the single-cell level using an adenovirus carrying *Per2-luc* (Fig. 1B). Performing quantification of signal intensity for each individual cell, which was visually distinguishable from other signals, revealed clear circadian rhythms in bioluminescence (Fig. 1C).

**Figure 2:**
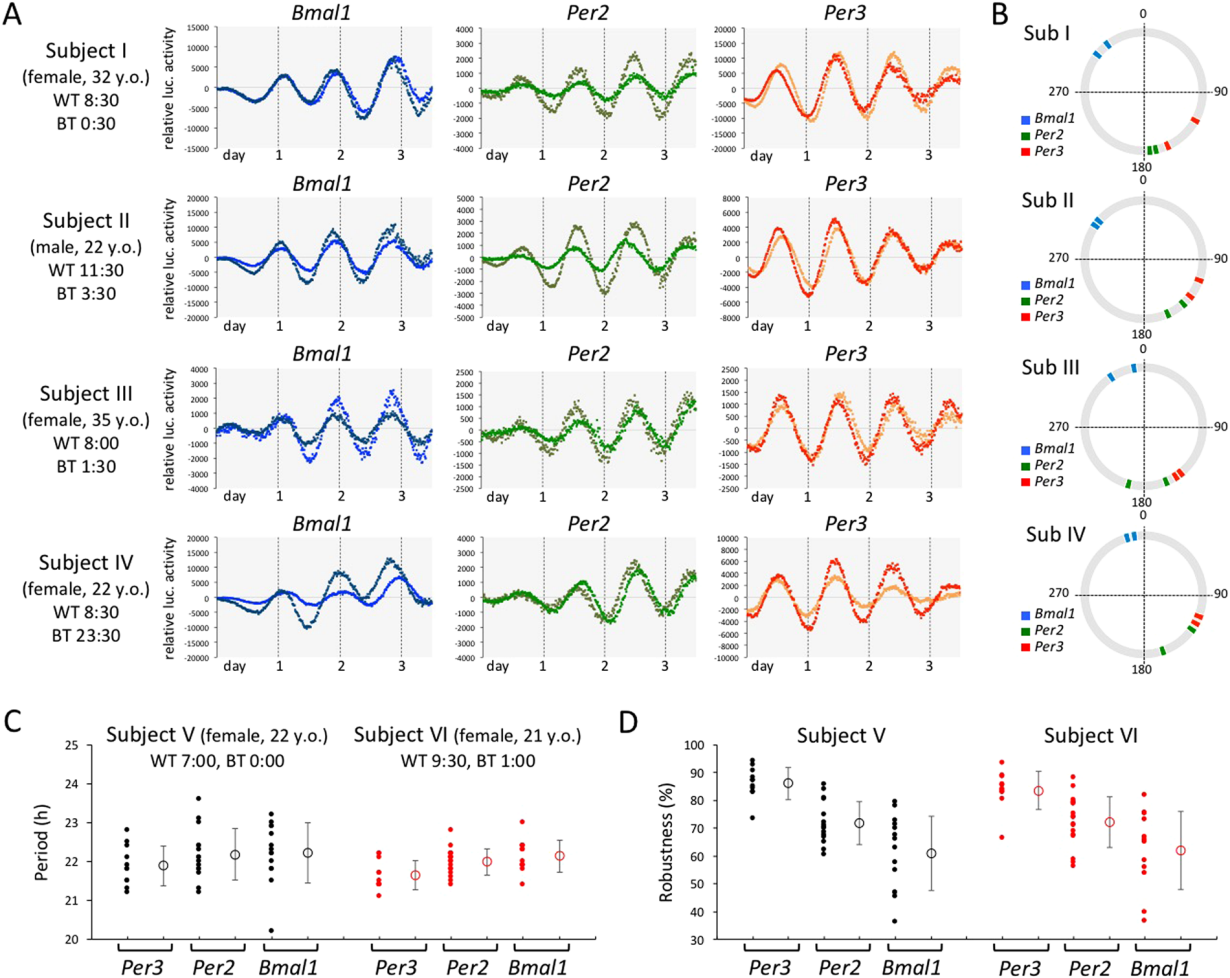
Phase relationship between bioluminescence rhythms of *Bmal1-luc*, *Per2-luc* and *Per3-luc.* (**A**) Hair roots obtained from four healthy subjects were infected with three adenovirus vectors carrying *Bmal1-luc*, *Per2-luc*, and *Per3-luc*. WT and BT indicate wake-up time and bedtime, respectively. Bioluminescence was measured in real time with a photomultiplier tube. To investigate reproducibility, two strands of hair were used for each virus vector (dark and light colored curves). Data sets were detrended by subtracting the 24-h running average from the raw data. (**B**) Phase angles of the data in (**A**) were calculated with a cosinor analysis and plotted on a circular graph. Oscillation data were considered reliable when both clear circadian oscillation persisted for ≥3 days and circadian robustness was 70% or more. (**C** and **D**) Period length (**C**) and robustness (**D**) were calculated with a cosinor analysis, and their average and standard deviation (SD) were compared among three adenovirus vectors in two subjects. Filled dots indicate the calculated period length and robustness for individual hair follicles, and open dots represent the mean ± SD.

Hair roots obtained from four healthy subjects were infected with three adenovirus vectors carrying *Bmal1-luc*, *Per2-luc*, and *Per3-luc*, respectively (Fig. 2A). Robust circadian oscillation in expression of three clock genes was detected as bioluminescence in all subjects. No obvious differences in circadian properties such as period length were observed, not only between subjects but also between hair roots. To facilitate understanding of the phase relationship between bioluminescence rhythms of *Bmal1-luc*, *Per2-luc* and *Per3-luc*, phase angles were calculated with a cosinor analysis and plotted on a circular graph (Fig. 2B). The phase of *Per2* expression rhythms is known to be slightly later than that of *Per3* and anti-phasic to that of *Bmal1* in multiple tissues *in vivo* ^19^, which was consistent with bioluminescence rhythms detected in all hair samples obtained from healthy subjects. As is qualitatively apparent, the luciferase activity driven by the *Per3* promoter was expected to represent the most stable and robust circadian rhythms, compared with the other adenovirus vectors. We therefore compared period length, robustness and standard deviation (SD) among three adenovirus vectors in two subjects (Fig. 2C and D). Although the average period length was almost identical, the SD of the *Per3* period length was consistently small. Additionally, *Per3* was the most robust at the smallest SD values. We therefore used the adenovirus vector carrying *Per3-luc* for subsequent experiments.

**Figure 3:**
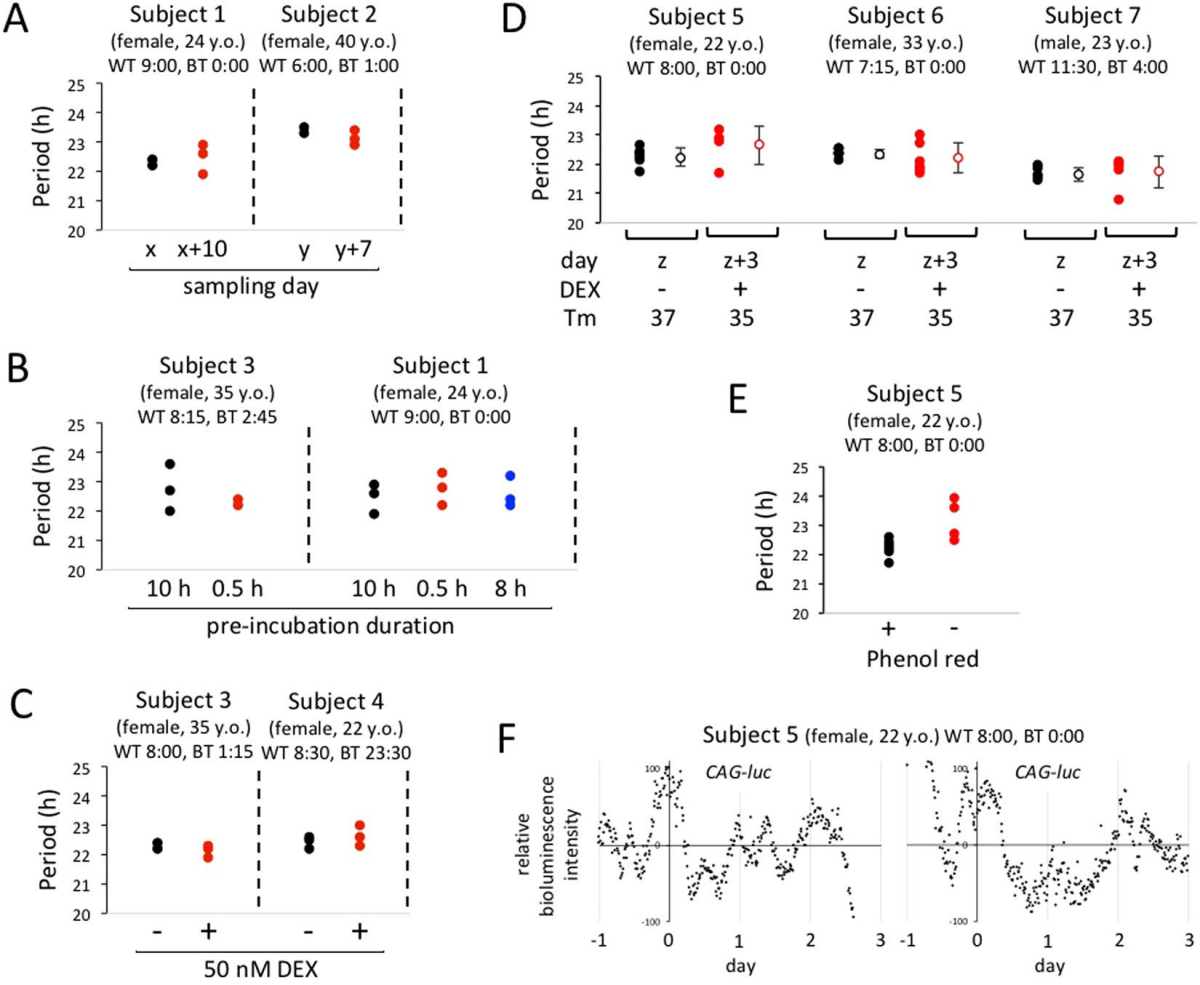
Validation of the *ex vivo* hairy root culture method. Hair roots obtained from healthy subjects were infected with an adenovirus vector carrying *Per3-luc*. WT and BT indicate wake-up time and bedtime, respectively. Bioluminescence was measured in real time with a photomultiplier tube. Data sets were detrended by subtracting the 24-h running average from the raw data, and the period length was calculated with a cosinor analysis. Oscillation data were considered reliable when both clear circadian oscillation persisted for ≥3 days and circadian robustness was 70% or more. The effect of difference in sampling day (**A**), the effect of difference in pre-incubation period (**B**), the effect of dexamethasone-induced synchronization of cellular clocks (**C**), the simultaneous effect of the difference in sampling day, measurement instrument, ambient temperature and DEX treatment (**D**), and the effect of the presence or absence of phenol red (**E**) were examined by comparing circadian period length between different conditions. To investigate reproducibility, two or three strands of hair were used for each experimental condition. (**F**) Hair roots were infected with a control adenovirus expressing the luciferase gene under regulation by the CAG promoter, a non-circadian promoter. Bioluminescence data sets were detrended by subtracting the 24-h running average from the raw data.

To confirm the reliability in evaluation of circadian properties based on *ex vivo* culture of whole hair root tissue, the stability and robustness of circadian period length in hair samples were investigated under different experimental conditions (Fig. 3). Specifically, we assessed the effect of difference in sampling day (Fig. 3A); the effect of difference in pre-incubation period, which was required, for example, for carrying samples from hospitals to laboratories (Fig. 3B); and the effect of dexamethasone-induced synchronization of cellular clocks (Fig. 3C), by comparing circadian period length in hair follicle cells between different conditions. A difference in sampling day of more than 1 week, difference in pre-incubation period of approximately 10 h, and dexamethasone treatment showed no obvious effect on circadian period length of autonomous clock gene expression. The reason why DEX treatment did not affect circadian period length may be due to the circadian entrainment caused by adenoviral infection prior to DEX administration. Next, we examined the simultaneous effect of the difference in sampling day, measurement instrument, ambient temperature and DEX treatment on *Per3* rhythms in cultured hair follicle tissue, and again confirmed that circadian period length was not significantly changed (Fig. 3D). Although almost all deduced period lengths were less than 24 h, which was inconsistent with human *in vivo* period length, this unexpected short period length was found to be at least partially dependent on culture medium composition (Fig. 3E). We compared circadian period length in hair follicle tissue in the presence or absence of phenol red, a pH indicator, and found that the lack of phenol red resulted in a rapid damping and relatively long period in bioluminescence rhythms. Phenol red is well known to have an estrogenic activity, and functional estrogen receptors are expressed in hair follicle tissue. Therefore, while phenol red may cause a shorter period length in hair follicle tissue, it may provide a better culture environment for continuous monitoring of bioluminescence rhythms. The effect of culture medium composition on circadian period length has been reported in a number of previous studies^20, 21^. Finally, we confirmed the absence of circadian oscillation of bioluminescence in hair follicle tissue infected with a control adenovirus expressing the luciferase gene under regulation by the CAG promoter, a non-circadian promoter (Fig. 3F)^13^.

### Correlation in circadian period length between clock gene expression in hair follicle tissue and locomotor activity

**Figure 4:**
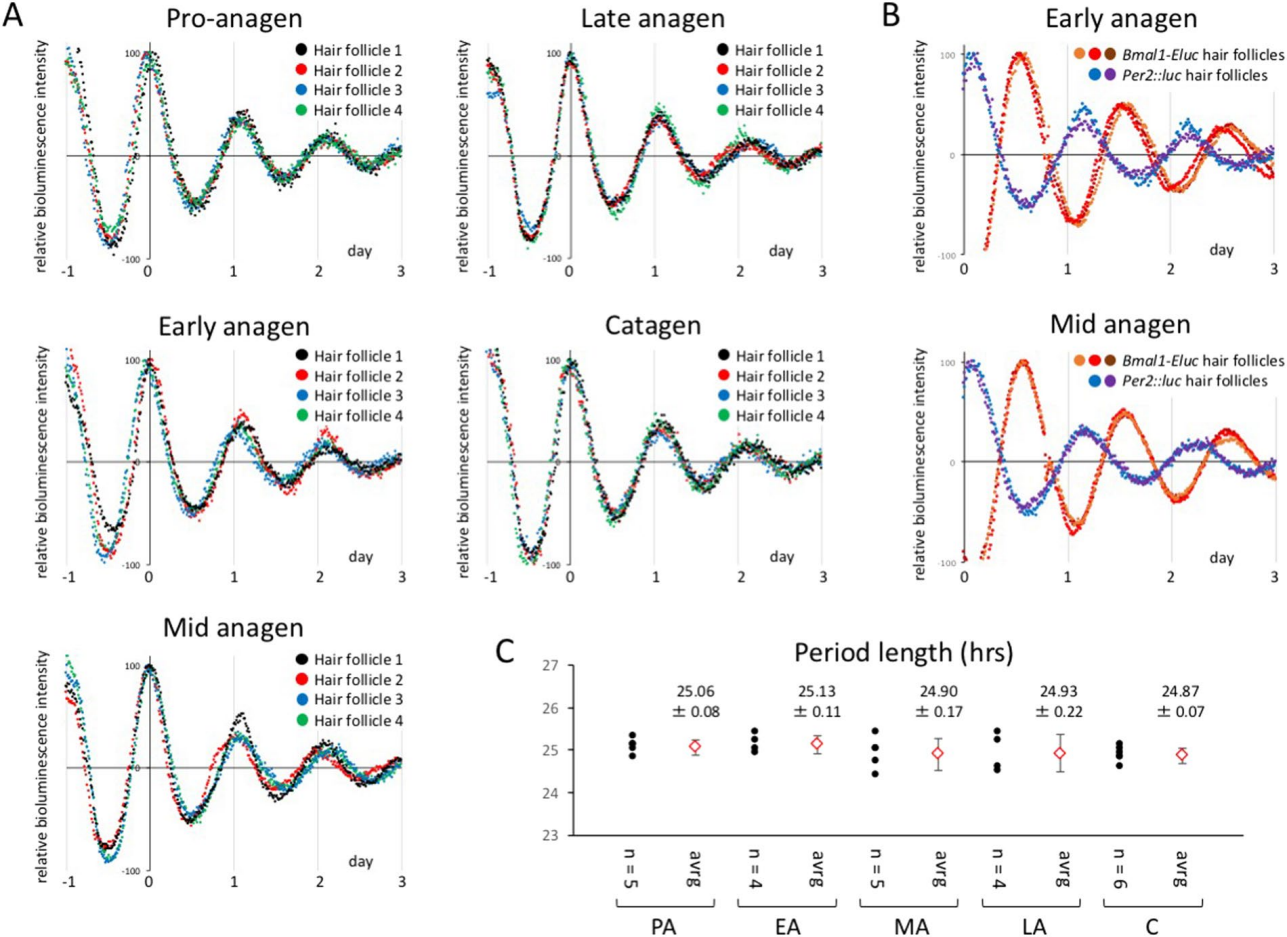
Comparison of circadian period length among hair cycle stages using mouse whisker follicles. (**A** and **B**) Whisker hair follicles were isolated by microdissection from a 6 week-old male *Per2* ^*Luc/*+^ mouse (**A**) and a 10 week-old male *Bmal1-Eluc* mouse (**B**). The hair cycle stage of whisker follicles was classified by morphology of the hair bulb and relative length of the hair shaft. The hair shaft was fixed on the bottom of a culture dish with silicone, and the immobilized hair tissue was then covered with luciferin-containing medium. After 2-day pre-culture, circadian synchronization was performed with 100 nM dexamethasone (DEX), and bioluminescence was measured in real time with photomultiplier tubes. Data sets were detrended by subtracting the 24 h running average from the raw data, and the first peak was then set to 100. Time 0 was defined as the time of the first peak (**A**) and the measurement start (**B**). The data are a representative of three independent experiments. (**C**) Period length calculated with a cosinor analysis. Black dots indicate the calculated period length of each hair follicle, and red dots represent the mean ± SD of period length values.

Although the results shown in Figs 2 and 3 indicate the possibility that human circadian period length can be easily estimated with high reproducibility using hair follicle tissue culture, estimated period length may vary among stages of the hair growth cycle, a process which results in cyclic and dynamic tissue remodeling. To exclude this possibility, we examined the difference in period length among hair stages using mouse whisker follicles, whose hair stages can be visually classified. Whisker follicles were isolated by microdissection from 5- to 6-week-old male heterozygous *Per2*::*luc* mice, and their hair cycle stage was classified as anagen or catagen by morphology of the hair bulb and relative length of the hair shaft. After 2-day pre-culture, circadian synchronization was performed with 100 nM dexamethasone (DEX), and then bioluminescence was measured in real time in the presence of luciferin with a photomultiplier tube (Fig. 4A). Hair follicles at all stages, including catagen, showed a clear circadian oscillation in bioluminescence. Additionally, consistent with the phase relation *in vivo*, circadian bioluminescence oscillation in hair follicles obtained from *Bmal1-Eluc* mice was antiphasic to that of *Per2*::*luc* mice (Fig. 4B). Moreover, we investigated the effect of the differences in hair stages on circadian period length (Fig. 4C), and found that there was no significant difference in circadian period length among hair stages.

**Figure 5:**
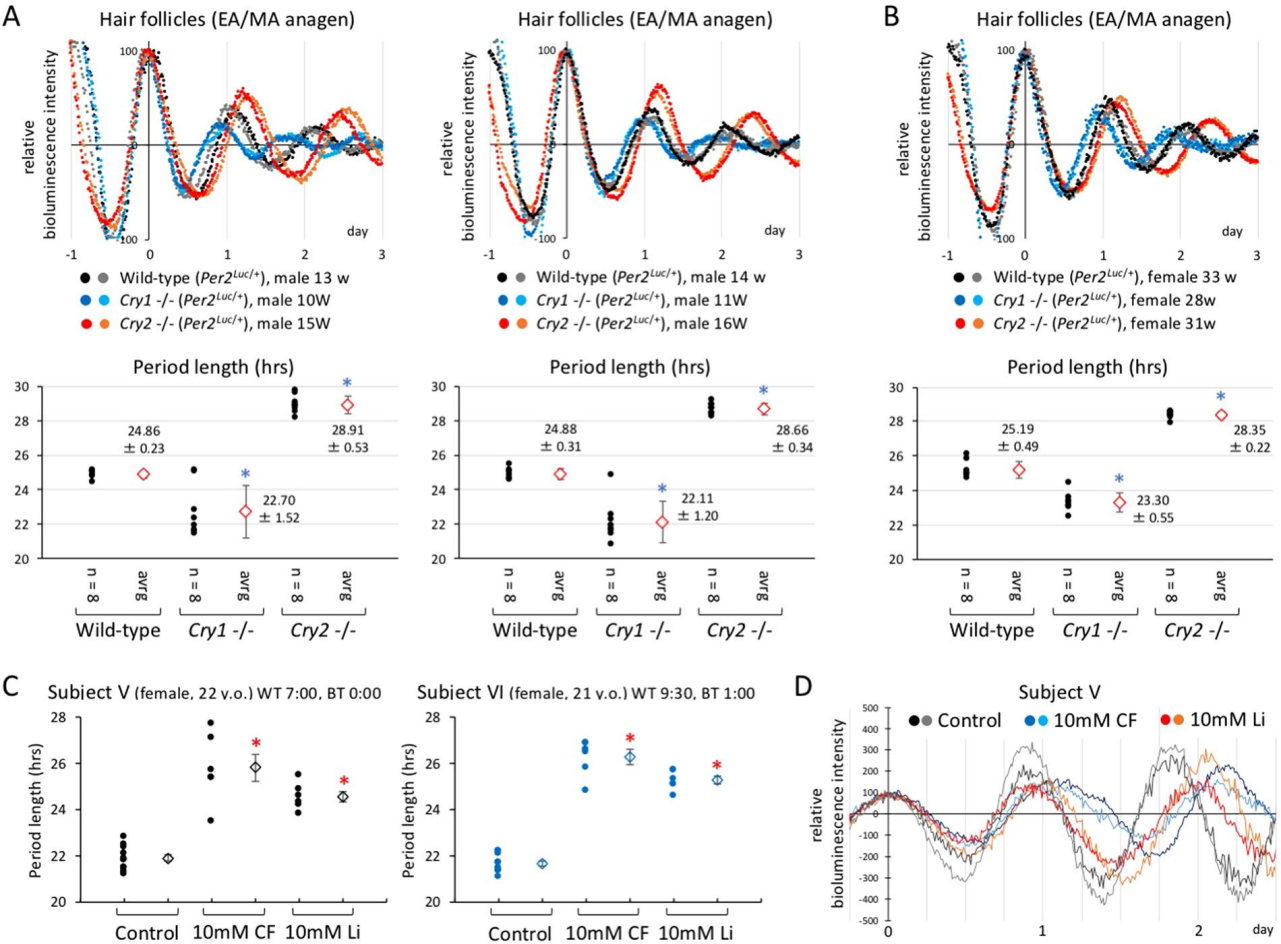
Comparison of circadian period length in whisker follicles among wild-type, *Cry1*- and *Cry2*-deficient mice. (**A** and **B**) Whisker hair follicles were isolated by microdissection from wild-type and knockout mice carrying the heterozygous *Per2*::*luc* allele. The hair cycle stage of whisker follicles was classified by morphology of the hair bulb and relative length of the hair shaft. (**A**) and (**B**) indicate the results obtained from three independent experiments (**A**, two sets of young male mice; **B**, middle-aged female mice). (Upper) After 2-day pre-culture, circadian synchronization was performed with 100 nM dexamethasone (DEX), and bioluminescence was measured in real time with photomultiplier tubes. Data sets were detrended by subtracting the 24 h running average from the raw data, and the first peak was then set to 100. Time 0 was defined as the time of the first peak. (Bottom) Period length calculated with a cosinor analysis. Black dots indicate the calculated period length of each hair follicle, and red dots represent the mean ± SD of period length values. Asterisks indicate a significant difference from wild-type (t test, P < 0.05). (**C** and **D**) The effect of 10 mM caffeine and lithium on the autonomous circadian oscillator in hair tissue from two human subjects. Chronic treatment with these reagents was started after removal of the adenovirus carrying *Per3-luc* from the culture medium. (**C**) Period length was calculated by cosinor analysis. Filled dots indicate the calculated period length of individual hair follicles, and open dots represent the mean ± SE. Asterisks indicate a significant difference compared to wild-type (t test, P < 0.05). (**D**) A representative set of bioluminescence data that were detrended by subtracting the 24 h running average from the raw data. The first peak was then set to 100. Time 0 was defined as the time of the first peak.

Many studies have shown a correlation in circadian period length between clock gene expression in peripheral tissue and physiology and behavior. In this study, to investigate whether circadian period length in hair follicle tissue reflects that in physiology and behavior, we used 10- to 16-week-old male *Cry1* and *Cry2* knockout mice carrying the heterozygous *Per2*::*luc* allele. Previous studies reported that *Cry1* and *Cry2* knockout mice showed a shorter and longer circadian period in locomotor activity, respectively, than wild-type mice^22^. Clear circadian oscillation in bioluminescence was detected in hair follicle tissue obtained from wild-type, *Cry1*- and *Cry2*-deficient mice, although a rapid damping was observed in *Cry1*-deficient mice (Fig. 5A and B, upper). Similar circadian patterns in bioluminescence were observed when using 28- to 33-week-old female mice. Moreover, we calculated circadian period length in cultured hair follicles and compared them among these genotypes, and found that *Cry1*- and *Cry2*-deficient mice showed a significantly shorter and longer circadian period, respectively, than wild-type mice (Fig. 5A and B, bottom). Although absolute values of circadian period length in cultured hair follicle tissue were different from those previously reported in locomotor activity, the relative difference in circadian period length among these genotypes was similar between clock gene expression in hair follicle tissue and locomotor activity. Given these results, circadian period length of clock gene expression in hair follicle tissue may correlate with intrinsic circadian period length observed in physiology and behavior.

To further confirm a correlation in circadian characteristics between clock gene expression in cultured hair follicle tissue and physiological or behavioral circadian rhythms at an individual level, we investigated whether the autonomous circadian oscillator in human hair tissue responded to caffeine and lithium in a similar manner to that at an individual level (Fig. 5C and D). Both these reagents are well known for lengthening the circadian period in mice^23–26^. We found that both these reagents significantly lengthened the circadian period of clock gene expression in cultured hair tissue from two human subjects. Previous studies suggest that caffeine or lithium administration phase-delays (transiently lengthens period) or lengthens the period, respectively, of human physiological circadian rhythms at an individual level^27, 28^, which is consistent with our present results in cultured hair tissue. Therefore, circadian characteristics in hair follicle tissue may correlate with those observed in physiology and behavior.

### Aging and dementia-resistant autonomous oscillation of peripheral clocks

To investigate the effect of aging and dementia on the human circadian clock machinery, old-old subjects with severe dementia, who showed abnormal circadian behavior, were recruited for analysis of circadian clock gene expression. The World Health Organization (WHO) defines the old-old as the people aged 75–84 and the oldest-old as 85+, although the definition varies among studies. We here classified subjects aged 75+ as the old-old. We did not consider sex differences because of the small sample size due to the difficulty of hair tissue collection from old-old dementia patients. The patients consisted of a man and four women, aged from 83 to 94 years. MMSE (Mini Mental State Examination) score was two or less in four of five patients, and FAST (Functional Assessment Staging) stage was seven in all patients. (also see Materials and Methods for detailed information about the patients). Because their daily time schedule was strictly controlled indoors, all patients were exposed to similar environmental cues before and during the experimental period.

**Figure 6:**
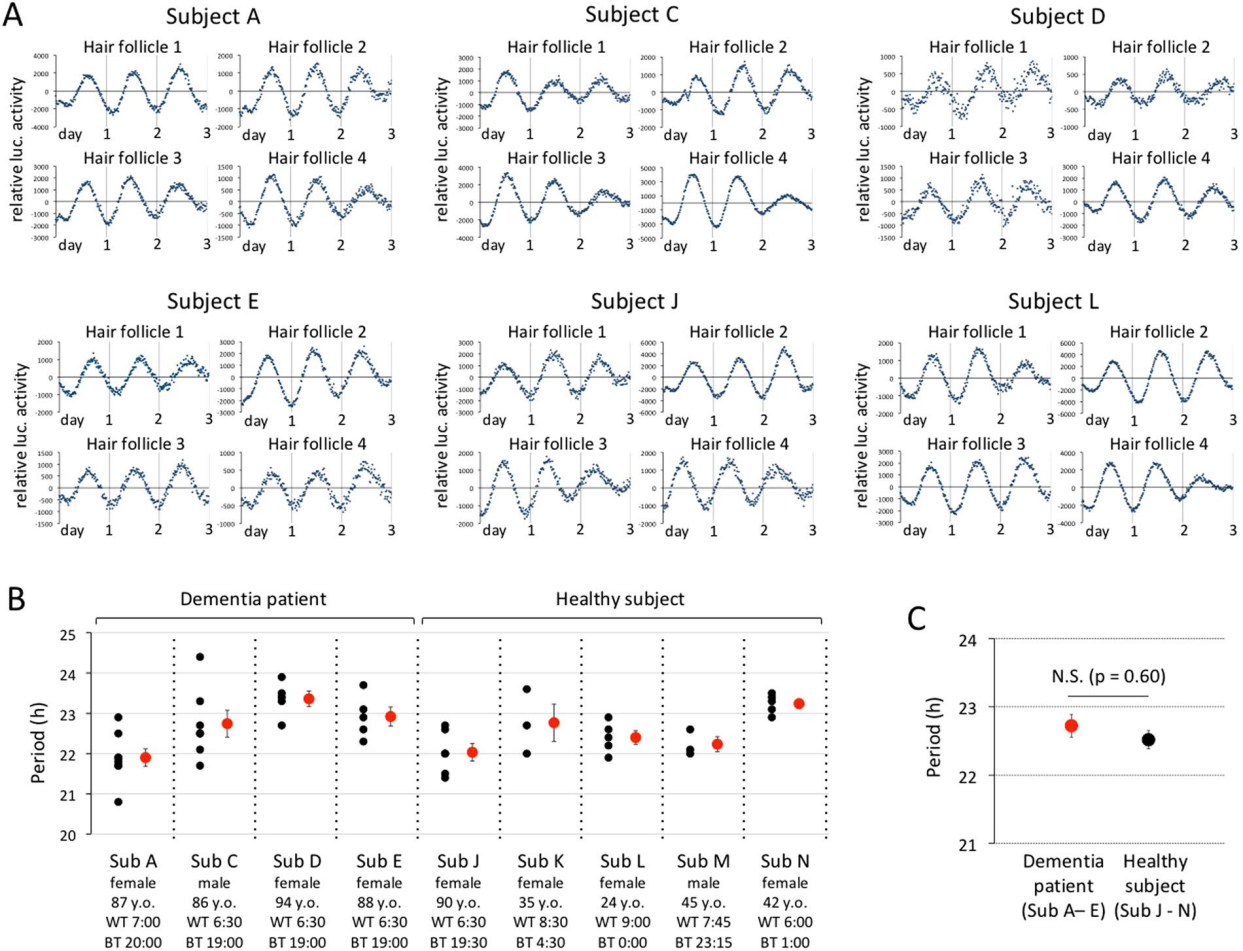
Comparison of bioluminescence rhythms in hair roots obtained from non-dementia subjects with those from dementia subjects. (**A**) Representative data obtained from hair roots of four dementia patients (**A**,**C**,**D** and **E**) and two healthy subjects (J and L) using an adenovirus vector carrying *Per3-luc*. Bioluminescence was measured in real time with a photomultiplier tube. To investigate reproducibility, four strands of hair were obtained from each subject. Data sets were detrended by subtracting the 24-h running average from the raw data. (**B**) Period length calculated with a cosinor analysis. Oscillation data were considered reliable when both clear circadian oscillation persisted for ≥3 days and circadian robustness was 70% or more. Black dots indicate the calculated period length of each hair follicle, and red dots represent the mean ± SE of period length values. (**C**) Student’s t-test for assessing the statistical difference in period length between healthy subjects and dementia patients. The average ± SE of period length was calculated using all period length values obtained from all subjects and patients in B (healthy subject, n = 22, 22.52 ± 0.13 h; dementia patient, n = 25, 22.72 ± 0.17 h). No statistically significant differences were detected between the two groups.

Cell-enriched hair roots obtained from old-old dementia patients (subjects A, C, D and E) were infected with an adenovirus carrying *Per3-luc*, and bioluminescence rhythms were monitored in real time (Fig. 6A). To avoid misestimation of circadian characteristics due to the heterogeneity of hair follicle tissue, we confirmed the reproducibility of the results by using multiple hairs. For comparison with healthy subjects, bioluminescence rhythms in hair roots obtained from two non-dementia women, aged 90 (subject J) and 24 (subject L) years, were measured under the same experimental conditions as dementia subjects’ samples. Bioluminescence data in both healthy subjects and patients showed similar circadian properties, indicating that cellular senescence and dementia pathology had no significant effect on autonomous oscillation performance in peripheral clock gene expression. To calculate peripheral period length, a cosinor analysis was performed for bioluminescence data obtained from cultured hair follicle cells of four patients and five healthy volunteers (Fig. 6B). Other than Subject J, healthy subjects were 45 or less years old. Calculated period length was considered to be reliable both when clear circadian oscillation persisted for 3 days or more and when circadian robustness was 70% or more. Although period plots indicated a significant difference in period length between individuals, no marked differences were noted between healthy subjects and dementia patients. Student’s t-test using all period length values obtained from all subjects and patients revealed no statistically significant differences between the two groups (Fig. 6C, healthy subjects, n = 22, 22.52 ± 0.13 h; dementia patients, n = 25, 22.72 ± 0.17 h).

## Discussion

Natural selection has favored endogenous circadian rhythmicity that autonomously persists with an intrinsic period close to that of Earth’s rotation in nearly all living organisms, including prokaryotes. In this study, to enable the investigation of intrinsic circadian characteristics in humans without using labor-intensive traditional experimental methods such as constant routine or forced desynchrony protocols, we developed a very simple and non-invasive method based on *ex vivo* culture of a whole hair root tissue. As shown in Fig. 5, circadian period length phenotypes in *Cry*-deficient mice and caffeine and lithium-induced changes in circadian period length indicate that circadian characteristics in cultured hair follicle cells reflect those at an individual level. Although further validation may be required to elucidate how correctly *ex vivo* circadian characteristics in hair root tissue correlates with that *in vivo*, this method enabled *ex vivo* evaluation of autonomous oscillation performance of clock gene expression and could be an approach for the estimation and evaluation of human intrinsic circadian characteristics.

This approach has been feasible using a method previously reported by Brown *et al*. based on human epidermal skin biopsy^9^. Importantly, those authors as well as others have concluded that circadian characteristics in peripheral cells such as skin-derived fibroblasts reflect individual circadian preference (chronotype). These interesting findings have been expected to make great contribution to the progress of human circadian biology. However, epidermal skin biopsy is too invasive for old or non-healthy subjects, and obtaining a sufficient number of fibroblasts for subsequent *in vitro* assays may be sometimes difficult, as cellular senescence attenuates fibroblast proliferation. In addition, cell dispersion or cell growth steps might potentially affect the original circadian characteristics. To compensate for these problems, we established a new method based on *ex vivo* culture of whole hair root tissue infected with adenovirus carrying the *luciferase* gene driven by circadian promoter/enhancer elements. This method exerts little burden on subjects, and the experimental procedure is extremely simple: pluck a few strands of facial or scalp hair, immerse them into a culture medium containing the adenovirus, and then place them into a device equipped with photomultiplier tubes.

However, the hair-tissue method has its own limitations: circadian period length in cultured hair tissue is slightly different from that *in vivo*, but this may be solved by adjusting the concentration of culture medium components, such as phenol red, as mentioned above (see Fig. 3E). Czeisler *et al*. reported that the human physiological circadian period is on average 24.18 hours in length in both young and old groups^29^. However, while Brown *et al*. reported a similar average circadian gene expression period of 24.5 h for fibroblasts, the circadian period of hair tissue grown in culture was more than one hour shorter than that those *in vivo* (healthy subjects, 22.52 h; dementia patients, 22.72 h). Nevertheless, this difference may not matter when comparing relative period length because the order relationship in period length among individuals is unchanged, as indicated in experiments using *Cry*-deficient mice in Fig. 5. The standard deviation of circadian period length among individuals is about 8 min for human physiology, about 45 min in cultured fibroblasts and about 31 min in cultured hair tissue^9^, suggesting that individual differences in circadian period length are enhanced under culture conditions. In addition to the limitation of short period length for the hair-tissue method, estimated circadian period length was unstable and variable among hair follicles when the *Bmal1* and *Per2* promoters were used. Consistent with this, in a previous report, we examined expression patterns of these three genes (*Per3*, *Bmal1* and *Per2*) in hair follicle tissue with realtime PCR, and found that only *Per3* showed clear circadian rhythms in mRNA levels with good reproduction^30^. Although PER3 may not be an indispensable component of the core clock machinery, as has been reported in previous reports, it should be a reliable circadian molecular marker because the phase interval among circadian expression of clock genes including *Per3* is nearly constant, not only in various tissues but also in cultured cells under different medium conditions^19^.

An age-related shortening of circadian period has been hypothesized to account for the circadian phase advance and early-morning awakening observed frequently in the elderly. To examine the effect of cellular senescence on autonomous circadian gene expression in humans, organisms with a much longer lifespan than that of model organisms such as mice, we performed *ex vivo* culture of a whole hair root tissue obtained from old-old dementia patients aged 83 to 94 with severe dementia who showed abnormal circadian behavior. Data obtained using this method showed that autonomous circadian oscillation performance in senescent cells obtained from dementia patients had no distinct abnormalities, suggesting no apparent effect of cellular senescence and dementia pathology on the autonomous clock oscillator machinery. As mentioned above, previous reports based on human epidermal skin biopsy have demonstrated that *in vitro* circadian period length in peripheral cells significantly correlates with individual human circadian preference^9, 10^. Nevertheless, an unexpected finding was the lack of any significant difference in the peripheral circadian period between healthy subjects and dementia patients with abnormal circadian behavior. On the other hand, this result is consistent with a report that *in vivo* circadian period length is nearly the same between younger and older subjects^29^.
